# Supplementary material for: Gene Expressions and High Lymphocyte Count May Predict Durable Clinical Benefits in Patients with Advanced Non-Small-Cell Lung Cancer Treated with Immune Checkpoint Inhibitors
Source: Cancers (Basel). 2023 Sep 8;15(18):4480. doi: 10.3390/cancers15184480 (PMC10526901; doi:10.3390/cancers15184480)
Supplement: Supplementary file 1 [file cancers-15-04480-s001.zip › Supplementary.pdf]

Figure S1. Swimmer plot displaying the ICI treatment trajectory and follow up for each patient.

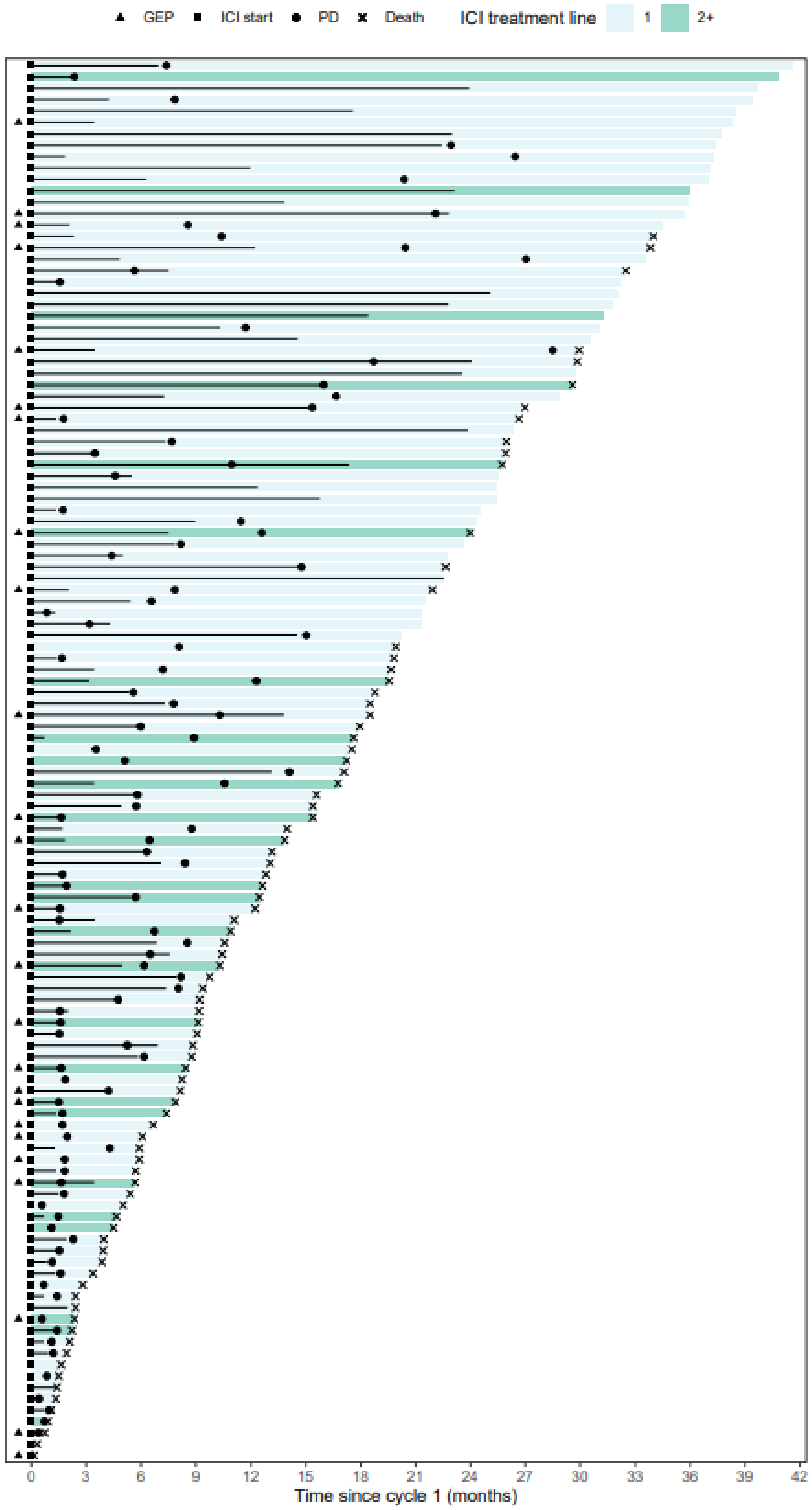

Patients who were included in the GEP cohort are marked with ▲

GEP, gene expression profiling; ICI, immune checkpoint inhibitor; PD, progressive disease

Figure S2. Univariable logistic regression analysis of factors associated with durable clinical benefit

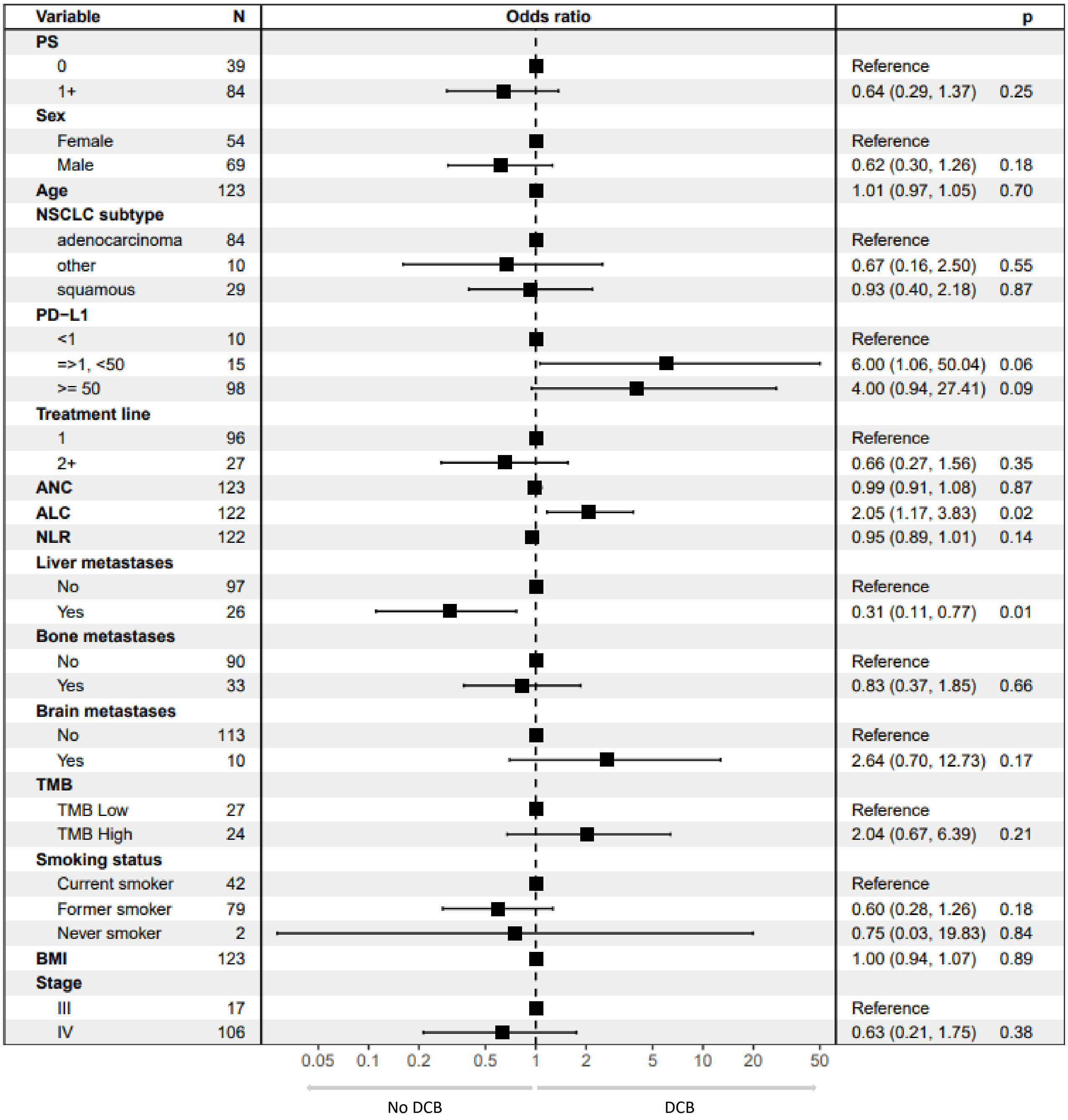

Variables with a p-value below 0.05 was considered significantly associated with durable clinical benefit (DCB). Wald test p-values and profile likelihood confidence limits are reported, which explain the non-significant p-value of 0.06 for PD-L1 ≥1 to <50% despite a confidence interval not including 1.

'Other' histopathology (NSCLC subtype) included NSCLC NOS and sarcomatoid carcinoma

N, number of patients; PS, performance status; NSCLC , non-small cell lung cancer; PD-L1, programmed death-ligand 1; ANC, absolute neutrophil count; ALC, absolute lymphocyte count; NLR, neutrophil-to-lymphocyte ratio; TMB, tumor mutational burden; BMI, body mass index

Figure S3. Association between absolute lymphocyte count (ALC) and durable clinical benefit (DCB)

a)

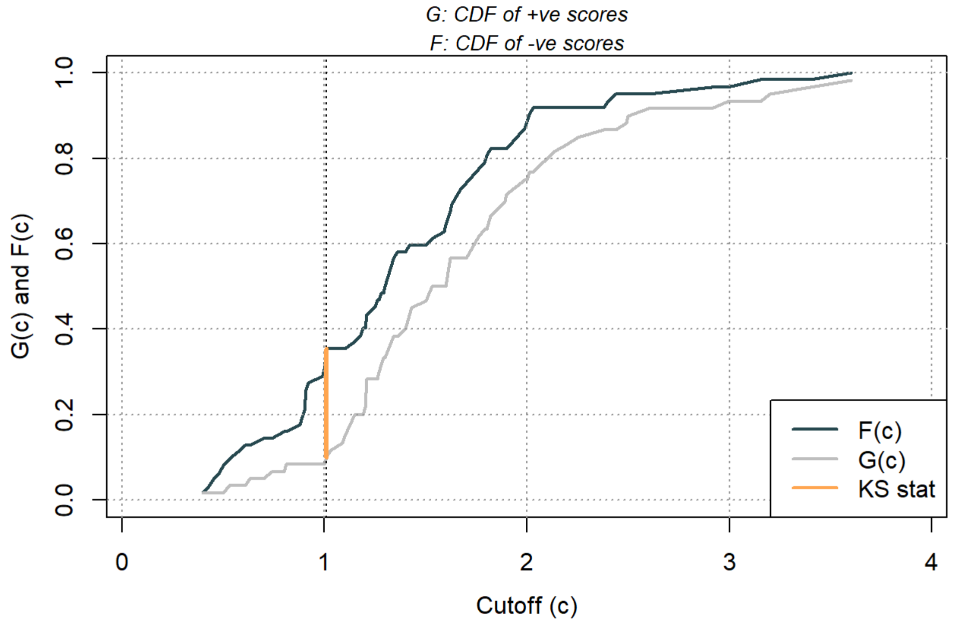

A Two-sample Kolmogorov–Smirnov plot was used to find the optimal ALC cut-off for predicting DCB. This optimal cut-off was determined as the cut-off value of the ALC that yielded the maximal difference between the cumulative density of ALC in the DCB positive/DCB negative group. The optimal ALC cut-off was 1.01 10^9/l.

b)

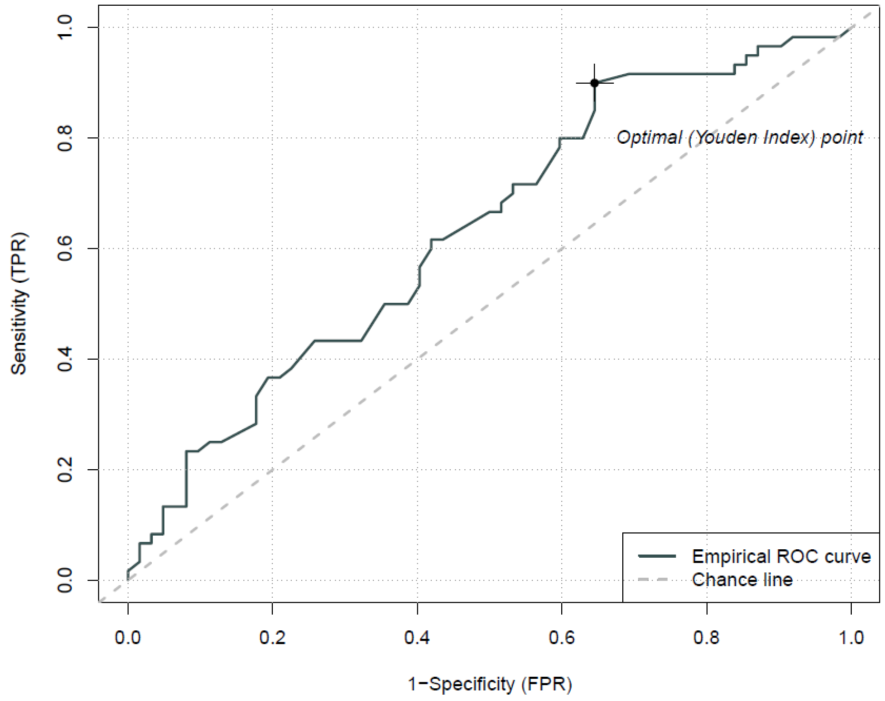

The cut-off (ALC 1.01 10^9/l) was used to dichotomize the ALC. A ROC curve using ALC as a predictor for DCB was drawn, and an AUC of 0.63 was found.

Figure S4. Multivariable Cox regression analysis of factors associated with overall survival in patients receiving 1L ICI treatment.

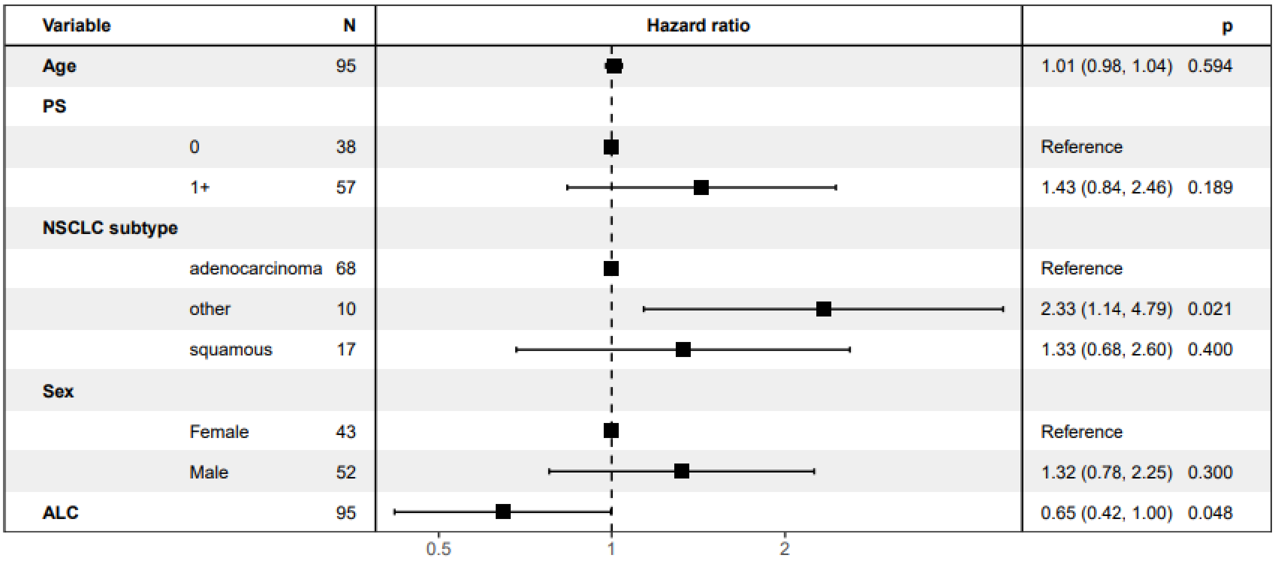

Variables with a p-value <0.05 was considered significantly associated with overall survival (OS).
'Other' histopathology (NSCLC subtype) included NSCLC NOS and sarcomatoid carcinoma
N, number of patients; PS, performance status; NSCLC, non-small cell lung cancer; ALC, absolute lymphocyte count

Table S1. Baseline characteristics and peripheral blood values according to treatment line

**Baseline characteristics**	**1L n (%)**	**≥2L n (%)**	**Total n (%)**	**p-value**
Patients	96 (78)	27 (22)	123 (100)	
Age, median years (range)	66 (46-86)	70 (52-83)	67 (46-86)	0.12
Sex				
Male	52 (54)	17 (63)	69 (56)	0.51
Female	44 (46)	10 (37)	54 (44)	
Performance status				
0	38 (40)	1 (4)	39 (32)	
1	48 (50)	16 (59)	64 (52)	<0.001
≥2	10 (10)	10 (37)	20 (16)	
Smoking status				
Current	30 (31)	12 (44)	42 (34)	
Former	64 (67)	15 (56)	79 (64)	0.37
Never	2 (2)	0 (0)	2 (2)	
BMI, median (range)	25 (16-41)	23 (18-40)	24 (16-41)	0.36
TNM stage				
III	14 (15)	3 (11)	17 (14)	0.76
IV	82 (85)	24 (89)	106 (86)	
Metastatic sites[a]				
Brain	8 (8)	2 (7)	10 (8)	1.0
Bone	28 (29)	5 (19)	33 (27)	0.33
Liver	18 (19)	8 (30)	26 (21)	0.29
Adrenal glands	23 (24)	7 (26)	30 (24)	0.81
Distant lymph nodes	9 (9)	7 (26)	16 (13)	0.05
Lung	19 (20)	14 (52)	33 (27)	0.002
Pleura[b]	35 (37)	8 (30)	43 (35)	0.65
Soft tissue[c]	5 (5)	0 (0)	5 (4.1)	0.59
Other	22 (23)	2 (7)	24 (20)	0.10
NSCLC subtype				
Adenocarcinoma	69 (72)	15 (56)	84 (68)	
Squamous cell carcinoma	17 (18)	12 (44)	29 (24)	0.008
Other[d]	10 (10)	0 (0)	10 (8)	
PD-L1				
<1%	0 (0)	10 (37)	10 (8)	
≥1% and <50%	1 (1)	14 (52)	15 (12)	<0.001
≥50%	95 (99)	3 (11)	98 (80)	
Blood values, median (range)*				
ALC (10^9/l)	1.42 (0.30-3.60)	1.27 (0.43-2.99)	1.40 (0.30-3.60)	0.33
ANC (10^9/l)	6.60 (2.90-36.3)	6.78 (3.14-16.2)	6.70 (2.90-36.3)	0.85
NLR	4.40 (1.16-34.7)	4.40 (1.99-37.7)	4.40 (1.16-37.7)	0.21

a) Patients could be registered with more than one metastatic site. Each metastatic site was recorded as a categorical variable (yes or no), and the p-values reflect the distribution of the two levels for each metastatic site.
b) 'Pleura' included pleural fluid
c) 'Soft tissue' included cutis, subcutis and muscles
d) 'Other' included NSCLC NOS (not otherwise specified) and sarcomatoid carcinoma
* ALC and NLR were missing in one patient treated with 1L ICI
1L, first-line ICI; ≥2L, second or subsequent line ICI; n, number of patients; BMI, body mass index; TNM, tumor-node-metastasis classification of malignant tumors; NSCLC, non-small cell lung cancer; PD-L1, programmed death-ligand 1; ALC, absolute lymphocyte count; ANC, absolute neutrophil count; NLR, neutrophil-to-lymphocyte ratio

Table S2. Treatment characteristics and clinical outcomes in patients with and without gene expression profiling

	**GEP n (%)**	**No GEP n (%)**	**Total n (%)**	**p-value**
Patients	25 (20)	98 (80)	125 (100)	
ICI treatment line				
1	15 (60)	81 (83)	96 (78)	0.03
≥2	10 (40)	17 (17)	27 (22)	
Days on treatment[a]				
Median (range)	56 (0-694)	139 (0-763)	105 (0-763)	0.11
Treatment beyond PD	3 (12)	8 (8)	11 (9)	0.69
ICI discontinuation due to[b]				
PD	11 (44)	57 (58)	68 (55)	0.26
Poor performance status	1 (4)	7 (7)	8 (7)	1
Two years of ICI	0 (0)	10 (10)	10 (8)	0.21
Toxicity	9 (36)	24 (25)	33 (27)	0.31
Death	1 (4)	3 (3)	4 (3)	1
Other reasons[c]	9 (36)	10 (10)	19 (15)	0.004
Subsequent treatment[d]	17 (68)	43 (44)	60 (49)	0.04
Clinical outcome				
DCB[e]	11 (44)	49 (50)	60 (49)	0.66
mOS, months (95% CI)	12.2 (0.2-38.3)	17.8 (0.3-41.7)	17.2 (0.2-41.7)	0.43

a) Median days on ICI treatment = time to treatment discontinuation
b) Each patient could be registered with more than one cause of ICI discontinuation
c) 'Other reasons' included lack of compliance, patient's choice, comorbidity, or high dose steroid
d) Patients receiving systemic antineoplastic treatment after ICI discontinuation. Only patients with a PD event during/after ICI treatment were recorded
e) DCB defined as PFS >6 months
GEP, gene expression profiling; ICI, immune checkpoint inhibitor; n, number of patients; PD, progressive disease; DCB, durable clinical benefit; mOS, median overall survival

Table S3. Differentially expressed genes between patients with DCB and without DCB with an unadjusted p-value <0.05

Gene	logFC	AveExpr	t	P.Value	adj.P.Val	B
LTBP1	-1.39	10.12	-4.44	0.0002	0.13	0.78
ITGAE	0.72	8.86	4.09	0.0004	0.16	0.06
COMP	-2.64	6.71	-3.87	0.0007	0.19	-0.52
HK1	0.98	10.72	3.59	0.0014	0.28	-1.02
ITGAX	-0.85	9.67	-3.32	0.0028	0.39	-1.56
ITGB3	-1.61	6.86	-3.14	0.0043	0.39	-1.94
FSTL3	-1.27	8.66	-3.14	0.0043	0.39	-1.93
SREBF1	0.95	10.83	3.14	0.0044	0.39	-1.94
TAP1	0.80	10.22	3.06	0.0053	0.39	-2.10
FGFR1	-0.95	8.13	-3.03	0.0057	0.39	-2.16
SIRPB2	-0.83	7.02	-3.02	0.0058	0.39	-2.17
SLC7A5	1.47	10.34	3.01	0.0059	0.39	-2.19
TMEM173	-0.79	10.54	-2.93	0.0072	0.44	-2.35
CX3CR1	-1.05	6.50	-2.80	0.0097	0.46	-2.59
BCL6B	-0.69	6.68	-2.80	0.0098	0.46	-2.59
MB21D1	-0.73	8.59	-2.79	0.0099	0.46	-2.61
CD1C	-1.37	5.60	-2.79	0.0100	0.46	-2.62
HMGA1	1.29	11.33	2.71	0.0122	0.51	-2.77
H2AFX	0.86	8.17	2.70	0.0123	0.51	-2.78
CSF1	-0.76	8.58	-2.65	0.0137	0.54	-2.88
CDH1	1.42	11.62	2.60	0.0155	0.57	-2.96
TP53	0.59	9.61	2.57	0.0165	0.57	-3.04
CLECL1	-1.02	6.05	-2.56	0.0171	0.57	-3.03
C5AR1	-0.72	6.65	-2.55	0.0175	0.57	-3.05
SOCS1	0.66	8.80	2.53	0.0183	0.57	-3.12
IFITM2	-0.69	11.20	-2.51	0.0190	0.57	-3.13
LILRA5	-0.67	7.15	-2.42	0.0231	0.64	-3.27
ITGAM	-0.74	7.31	-2.42	0.0232	0.64	-3.28
TGFB3	-0.83	8.67	-2.41	0.0236	0.64	-3.32
EZH2	0.77	8.52	2.40	0.0245	0.64	-3.35
SFXN1	0.91	9.94	2.38	0.0252	0.64	-3.37
CD274	1.11	8.38	2.34	0.0279	0.65	-3.45
MMP9	-1.02	8.11	-2.33	0.0281	0.65	-3.44
STAT1	0.61	12.39	2.33	0.0284	0.65	-3.44
EPCAM	1.18	10.76	2.28	0.0315	0.66	-3.54
SLC11A1	-0.80	7.81	-2.25	0.0339	0.66	-3.59
SERPINB5	1.57	9.29	2.24	0.0343	0.66	-3.59
LRRC32	-0.71	8.75	-2.22	0.0357	0.66	-3.65
VCAN	-1.03	12.04	-2.21	0.0366	0.66	-3.64

GPC4	-0.95	8.08	-2.19	0.0380	0.66	-3.68
TAP2	0.54	10.63	2.19	0.0384	0.66	-3.70
CD80	-0.73	7.16	-2.17	0.0395	0.66	-3.69
TPM1	-0.62	9.84	-2.17	0.0400	0.66	-3.75
ITGA4	-0.65	8.89	-2.14	0.0422	0.66	-3.79
TFRC	0.77	10.83	2.12	0.0442	0.66	-3.81
PRKAA2	0.87	6.17	2.11	0.0456	0.66	-3.78
GOT2	0.75	10.09	2.10	0.0461	0.66	-3.85
IFNG	0.87	5.29	2.10	0.0463	0.66	-3.77
CLEC5A	-1.09	6.95	-2.09	0.0470	0.66	-3.82
DAB2	-0.48	9.09	-2.09	0.0471	0.66	-3.88
SHC2	-0.89	7.68	-2.09	0.0475	0.66	-3.85
CD14	-0.62	9.67	-2.09	0.0475	0.66	-3.89
ERCC3	0.48	10.19	2.07	0.0491	0.66	-3.90

logFC: log2 fold change of DCB/No DCB
AveExpr: Average expression across all samples, in log2 CPM.
t: logFC divided by its standard error.
P.Value: Raw p-value (based on t) from test that logFC differs from 0.
adj.P.Val: Benjamini-Hochberg false discovery rate adjusted p-value.
B: log-odds that gene is differentially expressed (arguably less useful than the other columns).
